# Portal Hypertension Promotes Bacterial Translocation in Rats Mono- and Non Mono-Associated with Escherichia Coli C25

**DOI:** 10.1155/1994/57549

**Published:** 1994

**Authors:** Jean-Nicolas Vauthey, Petra Duda, Anthony M. Wheatley, Philippe Gertsch

**Affiliations:** 1 From the Department of Visceral and Transplantation Surgery University of Berne Inselspital Berne 3010 Switzerland; 2 University of Florida Department of Surgery P.O. Box 100286 Gainesville FL 32610 USA

## Abstract

The basis for the high incidence of infectious complications in portal hypertension (PHT) remains unclear. The hypothesis that PHT induces bacterial translocation (BT) was tested in a rat model with or without mono-association with streptomycin resistant Escherichia coli C25 and with or without hypovolemic shock. PHT was achieved by partial portal vein ligation and three weeks later hypovolemic shock (HS) was induced. Blood, liver, spleen and mesenteric lymph nodes cultures were performed twenty-four hours later.

PHT promoted BT to mesenteric lymph nodes in indigenous flora (4/6 [67%]) and mono-associated animals (7/9 [78%]) compared to sham laparotomy and sham shock (SL + SS) animals (0/6 [0%] and 2/9 [22%] respectively) (*p* = 0.03). The combination of PHT and HS resulted in increased mortality in mono-associated (7/15 [47%]) and non mono-associated animals (8/15 [53%]). No significant translocation was noted in liver and spleen and bacteremia was found only in the PHT + HS mono-associated animals (4/8 [50%]).

PHT induces BT to mesenteric lymph nodes and this may account for the high incidence of septic complications associated witti PHT. In this model, the addition of HS to PHT leads to an increased mortality but without uniform translocation of the gut flora beyond mesenteric lymph nodes.


*HPB Surgery*, 1994, Vol. 8, pp. 95–100
Reprints available directly from the publisher
Photocopying permitted by license only

© 1994 Harwood Academic Publishers GmbH
Printed in Malaysia

### Portal Hypertension Promotes Bacterial Translocation in Rats Mono- and Non Mono-Associated with Escherichia Coli C25

JEAN-NICOLAS VAUTHEY,* PETRA DUDA, ANTHONY M. WHEATLEY and PHILIPPE GERTSCH

From the Department of Visceral and Transplantation Surgery, University of Berne, Inselspital, 3010 Berne, Switzerland

The basis for the high incidence of infectious complications in portal hypertension (PHT) remains unclear. The hypothesis that PHT induces bacterial translocation (BT) was tested in a rat model with or without mono-association with streptomycin resistant Escherichia coli C25 and with or without hypovolemic shock. PHT was achieved by partial portal vein ligation and three weeks later hypovolemic shock (HS) was induced. Blood, liver, spleen and mesenteric lymph nodes cultures were performed twenty-four hours later.

PHT promoted BT to mesenteric lymph nodes in indigenous flora (4/6 [67%]) and mono-associated animals (7/9 [78%]) compared to sham laparotomy and sham shock (SL + SS) animals (0/6 [0%] and 2/9 [22%] respectively) ($p = 0.03$). The combination of PHT and HS resulted in increased mortality in mono-associated (7/15 [47%]) and non mono-associated animals (8/15 [53%]). No significant translocation was noted in liver and spleen and bacteremia was found only in the PHT + HS mono-associated animals (4/8 [50%]).

PHT induces BT to mesenteric lymph nodes and this may account for the high incidence of septic complications associated with PHT. In this model, the addition of HS to PHT leads to an increased mortality but without uniform translocation of the gut flora beyond mesenteric lymph nodes.

KEY WORDS: Portal hypertension bacterial translocation

### INTRODUCTION

Sepsis and infections remain the most serious complications of patients with liver cirrhosis and portal hypertension (PHT)[1]. The incidence of bacteremia in patients with bleeding esophageal varices (portal hypertension and shock) has been reported to be as high as 22% [2]. The bacteria isolated belong to the intestinal flora (Escherichia coli, Proteus mirabilis, Enterobacter, Bacteroides).

Although immunosuppression related to cirrhosis (changes in the reticuloendothelial system of the liver, complement, polymorphonuclear leucocyte and monocyte function impairment)[3,4,5] has been implicated as a contributing factor, the exact mechanism leading to infection in cirrhosis remains unknown. Recent data suggest that antimicrobial prophylaxis in cirrhotics with gastrointestinal hemorrhage can reduce the incidence of bacterial infections[6]. Other studies have shown that selective intestinal decontamination prevents the incidence of spontaneous bacterial peritonitis in cirrhotic patients[7].

---

**Present address*: Jean-Nicolas Vauthey, Assistant Professor, University of Florida, Department of Surgery, P.O. Box 100286, Gainesville, FL 32610, USA

Presented in part at the 43rd Annual Meeting of the American Association for the Study of Liver Disease in Chicago, November 1st, 1992

Supported by the Swiss National Foundation for Scientific Research (grant No 32-30009-90)

In the following experiment, the hypothesis that portal hypertension (PHT) *per se* could induce bacterial translocation (BT)—the passage of live bacteria across the intestinal barrier—was tested. In addition, as part of the same experiment, some animals were submitted to hypovolemic shock, a common complication of PHT. The consequences of the combination of shock and portal hypertension on bacterial translocation were analyzed.

### MATERIALS AND METHODS

Eighty-four male Wistar rats weighing 200–250 gm were investigated in a model combining portal hypertension (PHT) and/or hypovolemic shock (HS) with or without mono-association with streptomycin resistant Escherichia coli C25. The experimental design is illustrated in Figure 1. The study was approved by the Animal Ethics Committee for Canton Berne. The animals were maintained in individual cages and allowed food and water ad libitum. All operations were done under pentobarbital anesthesia (0.05 g/kg intraperitoneally) and sterility was maintained during the operative procedures.

PHT was induced by partial portal vein ligation (PPL) as described by Chojkier and Groszmann[8]. The technique consisted of ligation of the portal vein over a 21 gauge blunt needle placed above the confluence of the splenic and superior mesenteric veins with 4.0 non-absorbable suture. The needle was withdrawn immediately after ligation and patency of the portal vein was confirmed. Sham laparotomy (SL) consisted only of portal vein mobilization. Portal hypertension was studied in a pilot study including six PPL and 7 SL animals followed over three weeks. Body weight did not differ significantly between the two groups. PHT was confirmed by a rise in portal pressure in PPL rats (18.9 $\pm$ 3.7 mmHg) compared to SL rats (9.1 $\pm$ 3.2 mmHg) and verified by the presence of venous collaterals when the animals were sacrificed 21 days later.

In order to achieve bacterial mono-association, the indigenous intestinal flora was first cleared by adding bacitracin (0.4 mg/ml) and streptomycin (0.4 mg/ml) to the water from the fourteenth day on. Thereafter, all material in contact with the animals was sterilized (cages, food, water). On day eighteen, the animals were colonized by giving 100 ml of water containing an overnight culture of Escherichia coli C25.

Twenty days after PPL or SL the animals were anesthetized and the right femoral artery was cannulated. The rats were submitted to 70 minutes of hypovolemic shock (HS) or sham shock (SS) as described by Baker and Deitch[9]. HS was induced by withdrawing blood into a syringe containing 100 IU of heparin in 0.3 ml of saline until the systolic pressure reached 30 mmHg. Continuous hypotension was monitored with a pressure transducer (Statham P23XL, Spectramed, USA) connected to a dynagraph recorder. Additional blood was withdrawn as needed as soon as

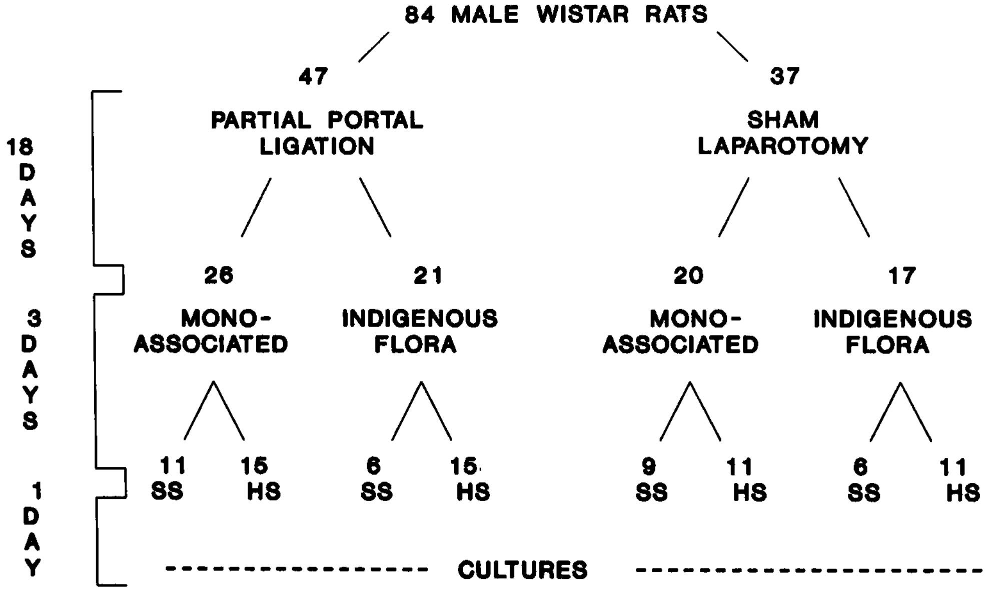

**Figure 1** Outline of the investigation. SS = Sham Shock, HS = Hypovolemic Shock.

the systolic blood pressure rose 10 mmHg or more. SS consisted of arterial cannulation and pressure measurement for 70 minutes. At the end of the HS, all shed blood was reinfused and one ml of blood was drawn for endotoxin measurements. Blood samples for endotoxins were centrifuged at 5000 g for 15 minutes, and the plasma was frozen immediately at −70°C for turbidimetric limulus amebocyte lysate analysis (LAL) (Pyroquant Diagnostik GmbH 6082 Walldorf, Germany) as previously described[10].

Twenty-four hours later the animals were killed. Blood samples from the portal vein and inferior vena cava were taken for endotoxin analysis. Five ml of caval blood were sampled for aerobic cultures (Organon Technika, Durham, NC). The paracolic mesenteric lymph nodes (MLN), spleen and left lobe of liver were weighed and 1 gm samples placed in sterile homogenizing tubes containing 5 ml of normal saline and homogenized with a sterile teflon plunger. Dilutions of the homogenized tissues were spread on agar plates and the cultures read 24 hours later. Selective agars for coliform bacteria (violet bile glucose agar, Fakola AG, Basel, Switzerland) and gram negatives (violet bile agar, Fakola AG) and blood agar were used for cultures in non mono-associated animals and McConkey's agar was used for E. coli mono-associated animals. In addition, one gram of cecal content was homogenized with 10 ml of normal saline in PHT + SS and SL + SS animals and quantitative cultures (colony forming units [CFU]) performed in order to provide a control culture of the endoluminal flora.

Morphologic alterations of the intestinal mucosa of the ileum and cecum were analyzed by light microscopy. The tissues were fixed in 4% formaldehyde, embedded in paraffin and stained with hematoxylin-eosin.

The following *variables* were studied:

a. mortality of hypovolemic shock
b. bacterial translocation, as determined by aerobic cultures of blood and homogenates of lymph nodes, spleen and liver
c. endotoxin, as determined by LAL-tests (endotoxin analysis) in the arterial blood at the end of shock, and in portal and systemic venous blood at the time of sacrifice in the mono-associated animals
d. morphologic microscopic alterations of ileal and cecal mucosa in mono-associated animals

For statistical analysis, mortality and BT were compared using Fisher exact test. Means were compared by ANOVA (analysis of variance) followed by Bonferonni–Dunn (all means) test. Results were expressed in means ± standard deviation. P values lower than 0.05 were considered significant.

### RESULTS

Mortality was significantly increased when HS was combined with PHT in mono-associated and indigenous flora animals (Table 1). Portal hypertension led to significant BT to mesenteric lymph nodes in both groups (Table 2). Positive blood cultures were found in PHT + HS (Gram + rods, Gram − rods, Corynebacterium species and yeasts) in the mono-associated group only (Table 3). Liver and spleen cultures remained sterile in non mono-associated animals while one animal in the PHT + SS mono-associated group had a positive spleen culture (E. coli).

Cecal cultures showed no difference in the number of Gram negative bacteria between SL and PHT groups

**Table 1** Mortality in mono- and non mono-associated animals

| | *PHT+SS* | *PHT+HS* | *SL+HS* | *SL+SS* |
|---|---|---|---|---|
| Non mono-associated | 0/6 | 8/15(53)[§] | 4/11(36) | 0/6 |
| Mono-associated | 2/11(18) | 7/15(47)* | 4/11(36) | 0/9 |

Percentages are indicated in parenthesis
§ $p = 0.03$ compared to PHT + SS and SL + SS
* $p = 0.02$ compared to SL + SS

**Table 2** Bacterial translocation in mesenteric lymph nodes in mono- and non mono-associated animals

| | *PHT+SS* | *PHT+HS* | *SL+HS* | *SL+SS* |
|---|---|---|---|---|
| Non mono-associated | 4/6(67)[§] | 5/7(71)[§] | 2/7(29) | 0/6 |
| Mono-associated | 7/9(78)* | 5/8(63) | 3/7(43) | 2/9(22) |

Percentages are indicated in parenthesis
§ $p = 0.03$ compared to SL + SS
* $p = 0.03$ compared to SL + SS

**Table 3** Positive blood cultures in mono- and non mono-associated animals

| | *PHT+SS* | *PHT+HS* | *SL+HS* | *SL+SS* |
|---|---|---|---|---|
| Non mono-associated | 1/6(17) | 0/7 | 2/7(29) | 1/6(17) |
| Mono-associated | 1/9(11) | 4/8(50)* | 2/7(29) | 0/9 |

Percentages are indicated in parenthesis
* $p = 0.03$ compared to SL + SS

**Table 4** Endotoxin concentration in arterial, portal and venous blood in mono-associated animals

| | *PHT+SS* | *PHT+HS* | *SL+HS* | *SL+SS* |
|---|---|---|---|---|
| Arterial† | 71 ± 84 | 1968 ± 2349 | 3206 ± 2883* | 176 ± 223 |
| Systemic# venous | 45 ± 107 | 69 ± 50 | 196 ± 160 | 103 ± 168 |
| Portal# | 33 ± 50 | 395 ± 993 | 103 ± 120 | 191 ± 350 |

* $p < 0.01$ compared to PHT + SS and SL + SS
† Immediately after hypovolemic shock
### 24 hours after hypovolemic shock
Values are expressed in pg/ml in indigenous flora animals ($2.1 \pm 3.7 \times 10^6$ CFU/gm versus $1.6 \pm 2.3 \times 10^6$ CFU/gm respectively). However, in the same group, the total bacterial count and the coliform count were significantly higher in the SL group compared to the PHT group ($6.9 \pm 9.2 \times 10^8$ CFU/gm versus $5.9 \pm 1.0 \times 10^7$ CFU/gm and $2.0 \pm 2.3 \times 10^6$ CFU/gm versus $1.6 \pm 2.9 \times 10^5$ CFU/gm respectively) ($p < 0.05$). In mono-associated animals, Escherichia coli cultures of cecal content showed no differences between SL and PHT groups ($4.1 \pm 6.0 \times 10^8$ CFU/gm and $2.2 \pm 1.4 \times 10^8$ CFU/gm respectively).

Significant endotoxemia was found in the arterial blood immediately after shock in SL + HS (Table 4). Endotoxins rose in PHT + HS animals too, but this rise was not significant. The mean endotoxin levels in the portal and systemic venous blood 24 hours later were comparable.

SL + SS and PHT + SS animals showed no change or minimal edema and hyperemia of the villi in the ileum (Figure 2) and slight submucosal inflammatory changes in the cecum. SL + HS and PHT + HS

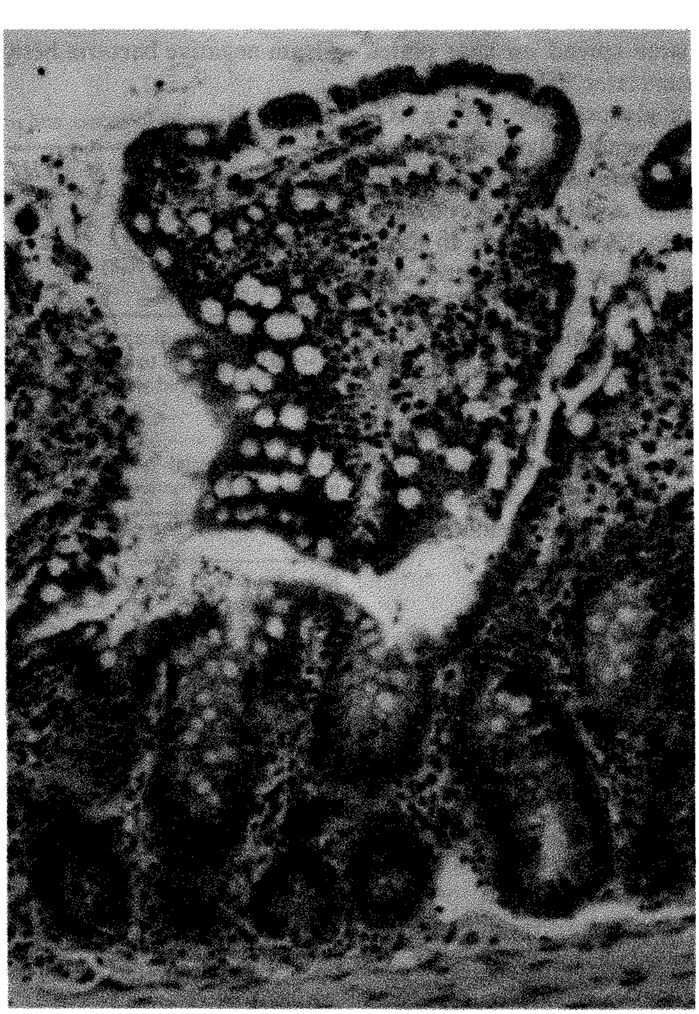

**Figure 2** Significant mucosal changes occurred only in the hypovolemic shock groups (SL + HS and PHT + HS) and included subepithelial edema and hyperemia of the villi of the ileum (240 × magnification).

animals showed diffuse moderate edema and hyperemia of the villi in the ileum and diffuse moderate edema with focally pronounced eosinophilic infiltrates in the submucosa of the cecum.

### DISCUSSION

Since Wolochow *et al.*[11] coined the term translocation to describe the passage of viable microorganisms from the gastrointestinal tract to the lymphatic system in rats, the importance of the interactions between the gut flora and the host have been increasingly recognized. Previous studies have demonstrated that bacterial translocation can be promoted by multiple factors including alteration of the indigenous flora[12], immunosuppression[13], severe injuries such as shock, trauma or burn[10,14], atrophy of the gut mucosa[15,16], endotoxemia[17] or sepsis [18].

Garcia–Tsao *et al.* have shown bacterial translocation forty-eight hours after PPL[19], but the present study demonstrates that chronic PHT promotes bacterial translocation to MLN. The animals maintained their initial weight and there were no physical signs of acute or chronic illness in the PHT group. A significant increase in portal pressure was observed immediately after PPL in the preliminary study and the development of porto-systemic collaterals attested to the presence of chronic portal hypertension. Previous studies have also shown that portal pressure remains elevated in rats after PPL in spite of the development of porto-systemic collaterals[20,21].

Speculations as to the mechanism by which PHT leads to bacterial translocation in this model remain open, but some causes can be excluded. In this experiment BT was tested in animals with indigenous flora and after mono-association with Escherichia coli C25 in order to control for possible variations in the gut flora between groups. There was significant BT to MLN in PHT animals whether or not these were associated with Escherichia coli C25.

In this investigation, no significant microscopic mucosal alterations account for the persistance of bacteria in MLN after three weeks. This finding results either from the persistence of BT to the MLN through a normal appearing mucosa or from defective clearance of the bacteria translocated immediately after PPL. Venous hypertension and possibly an alteration in the mesenteric lymphatic system might have contributed to this finding.

In a recent study on BT and PHT, almost 100% positive vena cava cultures were obtained in portal hypertensive rats 2 weeks following PPL[22]. In the present model of chronic PHT, no increased incidence of positive blood cultures was observed in PHT alone at 3 weeks. The present study suggests an effective systemic clearance of bacteria by the reticuloendothelial system including the liver and appears to confirm the clinical observation of rare positive systemic blood cultures in uncomplicated PHT[2]. In the clinical conditions of cirrhosis and ascites, impaired immune system[23] and hepatic functions[6] are important additional factors contributing to the high incidence of bacteremia and sepsis.

Previous studies have shown a high incidence of shock related bacteremia[9,22], but this study showed bacteremia only in mono-associated animals with PHT and HS. In the present study, the hypovolemic shock time was 70 minutes and shorter durations of shock were not studied. This led to a high mortality with only the most resistant animals being available for cultures twenty-four hours later. In addition, blood cultures performed after 24 hours may have been an underestimate of the true incidence of shock related bacteremia.

Significant endotoxemia was noted after HS only. The addition of PHT to HS seemed to be protective as the rise in endotoxin levels in this group was not significant. The absence of significant endotoxemia in the PHT group suggests that endotoxins did not contribute, at least on a chronic basis, to BT to MLN.

The finding that PHT induces BT to MLN provides further explanation for results of recent studies which showed obstructive jaundice[24] and hepatic resection[25] as unusual causes for BT. Recent experimental and clinical data suggest that these conditions are both associated with increased portal pressure[26–30], and it is likely that PHT contributed to BT in these investigations.

Quantitative studies will indicate whether the extent of MLN translocation correlates with the increase in portal pressure. Systematic MLN cultures should be undertaken to determine if BT also occurs in experimental models of ascites and cirrhosis[31]. Clinical studies confirming the exact significance of the PHT induced mesenteric bacterial reservoir and aiming at the prevention of translocation to the MLN are indicated.

### ACKNOWLEDGEMENTS

The authors thank Drs. Edwin A. Deitch[#] and Rodney D. Berg[†] (Departments of Surgery[#] and Immunology and Microbiology[†], Louisiana State Medical Center, Shreveport, Louisiana) for their helpful comments and suggestions and for providing the Escherichia coli C25 strain. The authors greatfully acknowledge Dr. Ch. Ruchti* and Dr. C. Breer** (Departments of Pathology* and Microbiology**, Inselspital, Berne, Switzerland) for their assistance. Dr. G. J. Maddern (Gastrointestinal Services Royal Adelaide Hospital, North Terrace, Adelaide South Australia, 5000) is acknowledged for his contribution to the initial protocol of investigation.

### REFERENCES

1. Wyke, R. J. (1989) Bacterial infections complicating liver disease. *Bail. Clin. Gastroenterol.*, **3**, 187–210.
2. Bleichner, G., Boulanger, R., Squara, P., Sollet, J. P. and Parent, A. (1986) Frequency of infections in cirrhotic patients presenting with acute gastrointestinal haemorrhage. *Br. J. Surg.*, **73**, 724–726.
3. Tanner, A. R., Arthur, M. J. P. and Wright, R. (1984) Macrophage activation, chronic inflammation and gastrointestinal disease. *Gut*, **25**, 760–783.
4. Such J., Guarner, C., Enriquez, J., Rodriguez, J. L., Seres, I. and Vilardell, F. (1988) Low C3 in cirrhotic ascites predisposes to spontaneous bacterial peritonitis. *J. Hepatology*, **6**, 80–84.
5. Chuang, W. L., Liu, H. W., Chang, W. Y., Chen, S. C., Hsieh, M. Y. and Wang L. Y. (1991) Natural killer cell activity in patients with liver cirrhosis relative to severity of liver damage. *Dig. Dis. Sc.*, **36**, 299–302.
6. Pauwels, A., Mostefa-Kara, N., Debenes, B., Degoutte, E., Florent, C. and Lévy, V. G. (1992) Antimicrobial prophylaxis after gastrointestinal hemorrhage for cirrhotic patients with a high risk of infection. Hepatology, **16**, 123A
7. Soriano, G., Guarner, C. and Teixidó, M., *et al.* (1991) Selective intestinal decontamination prevents spontaneous bacterial peritonitis. Gastroenterology, **100**, 477–481.
8. Chojkier, M., Groszmann, R. J. (1981) Measurement of portal-systemic shunting in the rat by using $y$-labeled microspheres. *Am. J. Physiol.*, **240**, G371–G375.
9. Baker, J. W., Deitch, E. A., Li, M., Berg, R. D. and Specian, R. D. (1988) Hemorrhagic shock induces bacterial translocation from the gut. *J. Trauma*, **28**, 896–906.
10. Fulenwider, J. T., Sibley, C., Stein, S. F., Evatt, B., Nordlinger, B. M. and Ivey, G. L. (1980) Endotoxemia of cirrhosis: an observation not substantiated. *Gastroenterology*, **78**, 1001–1004.
11. Wolochow, H., Hildebrand, G. J. and Lamanna, C. (1966) Translocation of microorganisms across the intestinal wall of the rat: effect of microbial size and concentration. *J. Infect. Dis.* **116**, 523–528.
12. Berg, R. D., Wommack, E. and Deitch, E. A. (1988) Immunosuppression and intestinal bacterial overgrowth synergistically promote bacterial translocation. *Arch. Surg.*, **123**, 1359–1364.
13. Alverdy, J. and Aoys, E. (1991) The effect of glucocorticoid administration on bacterial translocation. *Ann. Surg.*, **214**, 719–723.
14. Fukushima, R., Gianotti, L., Alexander, J. W. and Pyles, T. (1992) The degree of bacterial translocation is a determinant factor for mortality after burn injury and is improved by prostaglandin analogs. *Ann. Surg.*, **216**, 438–445.
15. Deitch, E. A., Ma, W. J., Ma, L., Berg, R. D. and Specian, R. D. (1990) Protein malnutrition predisposes to inflammatory-induced gut-origin septic states. *Ann. Surg.*, **211**, 560–568.
16. Alexander, J. W. (1990) Nutrition and translocation. *J.P.E.N.*, **14**, 170S–174S.
17. Deitch, E. A., Taylor, M., Grisham, M., Ma, L., Bridges, W. and Berg, R. (1989) Endotoxin induces bacterial translocation and increases xanthine oxidase activity. *J. Trauma*, **29**, 1679–1683.
18. Jones, W. G. II, Barber, A. E., Minei, J. P., Fahey, T. J. III. Shires, G. T. III, Shires, G. T. (1991) Differential pathophysiology of bacterial translocation after thermal injury and sepsis. *Ann. Surg.*, **214**, 24–30.
19. Garcia-Tsao, G., Albillos, A., West, A. B., Barden, G. E. (1991) Bacterial translocation in portal hypertension (PTH). *Hepatology*, **14**, 88A.
20. Vorobioff, J., Bredfeldt, J. E. and Groszmann, R. J. (1983) Hyperdynamic circulation in a portal-hypertensive rat model: a primary factor for maintenance of chronic portal hypertension. *Am. J. Physiol.*, **244**, G52–57.
21. Benoit, J. N., Womack, W. A., Hernandez, L., Granger, D. N. (1985) "Forward" and "backward" flow mechanisms of portal hypertension. Relative contributions in the rat model of portal vein stenosis. *Gastroenterology*, **89**, 1092–1096.
22. Sorell, W. T., Quigley, E. M. M., Jin, G., Johnson, T. J. and Rikkers, L. F. (1993) Bacterial translocation in the portal-hypertensive rat: studies in basal conditions and on exposure to hemorrhagic shock. Gastroenterology, **104**, 1722–1726.
23. Rajkovic, I. A. and Williams, R. (1986) Abnormalities of neutrophil phagocytosis, intracellular killing and metabolic activity in alcoholic cirrhosis and hepatitis. *Hepatology*, **6**, 252–262.
24. Deitch, E. A., Sittig, K., Li, M., Berg, R. and Specian, R. D. (1990) Obstructive jaundice promotes bacterial translocation from the gut. *Am. J. Surg.*, **159**, 79–84.
25. Wang, X., Andersson, R., Soltesz, V. and Bengmark, S. (1992) Bacterial translocation after major hepatectomy in patients and rats. *Arch. Surg.*, **127**, 1101–1106.
26. Franco, D., Gigou, M., Szekely, A. M. and Bismuth, H. (1979) Portal hypertension after bile duct obstruction. *Arch. Surg.*, **114**, 1064–1067.
27. Lebrec, D., Blanchet, L. (1985) Effect of two models of portal hypertension on splanchnic organ blood flow in the rat. *Clin. Sc.*, **68**, 23–28.
28. Zimmermann, H., Reichen, J., Zimmermann, A., Sägesser, H., Thenisch, B. and Höflin, F. (1992) Reversibility of secondary biliary fibrosis by biliodigestive anastomosis in the rat. *Gastroenterology*, **103**, 579–589.
29. Kanematsu, T., Takenaka, K., Furuta, T., Ezaki, T., Sugimachi, K. and Inokuchi, K. (1985) Acute portal hypertension associated with liver resection. *Arch. Surg.*, **120**, 1303–1305.
30. Lee, S. S., Hadengue, A., Girod, C., Braillon, A. and Lebrec, D. (1987) Reduction of intrahepatic vascular space in the pathogenesis of portal hypertension. *Gastroenterology*, **93**, 157–161.
31. Runyon, B. A., Sugano, S., Kanel, G. and Mellencamp, M. A. (1991) A rodent model of cirrhosis, ascites, and bacterial peritonitis. *Gastroenterology*, **100**, 489–493.

